# Prediction of reaction knockouts to maximize succinate production by *Actinobacillus succinogenes*

**DOI:** 10.1371/journal.pone.0189144

**Published:** 2018-01-30

**Authors:** Ambarish Nag, Peter C. St. John, Michael F. Crowley, Yannick J. Bomble

**Affiliations:** 1 Computational Science Center, National Renewable Energy Laboratory, Golden, Colorado, United States of America; 2 Biosciences Center, National Renewable Energy Laboratory, Golden, Colorado, United States of America; University of Huddersfield, UNITED KINGDOM

## Abstract

Succinate is a precursor of multiple commodity chemicals and bio-based succinate production is an active area of industrial bioengineering research. One of the most important microbial strains for bio-based production of succinate is the capnophilic gram-negative bacterium *Actinobacillus succinogenes*, which naturally produces succinate by a mixed-acid fermentative pathway. To engineer *A*. *succinogenes* to improve succinate yields during mixed acid fermentation, it is important to have a detailed understanding of the metabolic flux distribution in *A*. *succinogenes* when grown in suitable media. To this end, we have developed a detailed stoichiometric model of the *A*. *succinogenes* central metabolism that includes the biosynthetic pathways for the main components of biomass—namely glycogen, amino acids, DNA, RNA, lipids and UDP-N-Acetyl-α-D-glucosamine. We have validated our model by comparing model predictions generated via flux balance analysis with experimental results on mixed acid fermentation. Moreover, we have used the model to predict single and double reaction knockouts to maximize succinate production while maintaining growth viability. According to our model, succinate production can be maximized by knocking out either of the reactions catalyzed by the PTA (phosphate acetyltransferase) and ACK (acetyl kinase) enzymes, whereas the double knockouts of PEPCK (phosphoenolpyruvate carboxykinase) and PTA or PEPCK and ACK enzymes are the most effective in increasing succinate production.

## Introduction

Succinate is an important bio-based platform chemical and intermediate that can be converted to multiple commodity chemicals: namely 1,4-butanediol, tetrahydrofuran and γ-butyrolactone [1]. Succinate is used to produce the biodegradable plastic polybutylene succinate by heteropolymerization with 1,4-butanediol [2]. It can also be utilized to synthesize useful chemicals like ethylenediamine disuccinate, a biodegradable chelating agent, and diethyl succinate that can be utilized as a green solvent in lieu of methylene chloride [3]. Succinate also exhibits similarity in chemical structure to maleic anhydride, and therefore has the prospect of replacing this compound’s traditional petrochemical market [2]. Besides being based on renewable resources, bio-based succinate production has the advantage of being environmentally friendly since it utilizes CO_2_, a greenhouse gas, as a substrate [1, 4]. All bio-based succinate production involves fermentation by a variety of both wild and genetically modified bacterial strains.

The most prominent wild type bacterial strain for succinate production is the capnophilic and facultatively anaerobic, gram-negative bacterium *Actinobacillus succinogenes* [5], which naturally produces succinate by a fermentative pathway [3, 4]. This bacterial strain was first isolated from the bovine rumen as part of a search for succinogenic bacteria [6, 7]. This organism is capable of growth on most naturally occurring sugars [2, 6]. In addition to producing some of the highest reported succinate concentrations, this strain also yields significant amounts of formate and acetate [8]. The distribution of the carbon flux between succinate and other alternate fermentation products is influenced by experimental and environmental conditions [6]. Thus, increasing the available CO_2_ concentration is conducive to a higher succinate yield. Supplying a reductant e.g. H_2_ or utilization of carbon sources more reduced than glucose can also lead to higher amounts of succinate produced [6, 9]. One active research area in microbial succinate production is to produce succinate as the sole product of fermentation. McKinlay et al. have noted that optimizing the environmental conditions cannot suffice for homosuccinate fermentation [6]. Thus, genetic engineering of wild type *A*. *succinogenes* is necessary to produce succinate as the major product of mixed acid fermentation. It is of utmost importance to understand the metabolic flux distribution in *A*. *succinogenes* in order to effectively engineer the organism to enhance the succinate yield. One of the most useful tools to understand the metabolic flux distribution in any organism is computational modeling of the metabolic network [10–13] in the organism and constraint-based analysis of the metabolic network model [14, 15]. *A*. *succinogenes* has been widely studied experimentally, however, few metabolic models for *A*. *succinogenes* have been published.

Here, we describe the development of a metabolic model for *A*. *succinogenes* comprising 375 reactions. This model is intermediate in scale between a central carbon metabolism and a genome-scale metabolic model. While the model of Rafieenia [16] and other existing models proposed by McKinlay et al. [3, 4, 6, 8] focus on the central carbon metabolism, our model includes pathways for the major biomass components including amino acids, RNA, DNA, glycogen and lipids. Thus, our model can be termed as a comprehensive carbon metabolism model and not a genome scale model, since we do not explicitly include all the known metabolic pathways in *A*. *succinogenes*. Although a genome-scale model is more comprehensive than a central carbon metabolic model and is likely to have more predictive power, it is also associated with higher complexity and hence, requires significantly higher computational power [17]. On the other hand, most of the useful bio-chemicals and biofuel precursors that are of industrial importance are produced by the central carbon metabolism [18] and hence, from a synthetic biology/bioengineering perspective, it is sufficient to focus on the central carbon metabolism, rather than the genome-scale metabolism. We validate our model by comparison of the model predictions with extensively documented experimental results on *A*. *succinogenes* [3, 4, 6, 8]. We then use the model to make possible reaction knockout predictions to maximize succinate production. Based on the model predictions for the *in silico* wild type and mutant strains, we gain considerable insight into the carbon flow in the *A*. *succinogenes* metabolic network.

## Materials and methods

### Metabolic modeling

There are several computational methods that involve the use of a metabolic network and the assumption of a pseudo-steady state. These methods collectively constitute a framework commonly referred to as stoichiometric modeling [19]. This framework involves the estimation of the metabolic flux distribution using constraint-based analyses. Stoichiometric modeling bypasses the difficulties that arise in the development of kinetic models due to the lack of intracellular experimental measurements. Thus, the stoichiometric modeling framework enables the utilization of the knowledge of the structure and topology of the cell metabolic network, without having to depend on the largely incomplete knowledge of intracellular kinetics. Stoichiometric models have been used to estimate the cellular metabolic flux distribution under given circumstances, at a given time (metabolic flux analysis [20–23]), to predict the cellular metabolic flux distribution on the basis of some optimality hypothesis (flux balance analysis [24, 25]) and as tools for the structural analysis of metabolism to garner information about cellular systemic characteristics (network-based pathway analysis [26]). All these different methods require a metabolic network model. We have used both COBRApy [27] and the MATLAB COBRA Toolbox version 2.0.6 for the analyses described in this article [28].

#### Model development and validation

An automatically generated COBRA-compliant SBML model for *Actinobacillus succinogenes* (strain ATCC 55618/130Z) metabolism was downloaded from the BioModels database (http://www.ebi.ac.uk/biomodels-main/iID000000140364). However, this model was uncurated and could not yield finite biomass production when subjected to flux balance analysis (FBA). The KEGG database has curated *A*. *succinogenes* pathways and an uncurated (Tier 3) Pathway Genome Database for *A*. *succinogenes* can be accessed through the BioCyc database. We utilized these two resources, along with the above-mentioned genome-scale SBML model, and a wide body of experimental literature on *A*. *succinogenes* to develop and curate a working comprehensive carbon metabolic model of *A*. *succinogenes*. The biomass components that we considered in our model are i) the amino acids, ii) RNA, iii) DNA, iv) glycogen, v) lipids and vi) UDP- N-Acetyl-α-D-glucosamine (UDP-GlcNAc), which is an essential precursor of peptidoglycan and lipopolysaccharide.

We ensured that known mechanisms of uptake and excretion of various metabolites that have been experimentally reported were accurately encoded in the model. Notable among these mechanisms were PTS or ATP mediated uptake of sugars (6). For those metabolites for which the uptake/secretion mechanism was unknown we used the same mechanism as in *Escherichia coli* unless contradicted by published literature.

Experimental findings by McKinlay et al. [6] indicate that *A*. *succinogenes* is auxotrophic with respect to L-cysteine, L-glutamate and L-methionine. Hence, uptake reactions for these metabolites were incorporated into the model. *A*. *succinogenes* has an incomplete TCA cycle with the isocitrate dehydrogenase genes missing from its genome sequence. Hence, α-ketoglutarate, which is a L-glutamate precursor, cannot be synthesized from glucose via the TCA cycle, resulting in L-glutamate auxotrophy. We ascertained that the TCA cycle in our model was incomplete and that our model did not include a glyoxylate shunt in accordance with the experimental findings of McKinlay et al. [6, 8]. We also ensured that gene-protein-reactions associations in the model are consistent with KEGG and BioCyc databases on *A*. *succinogenes*.

We ensured our model was in a valid SBML format using http://sbml.org/validator/. We then checked whether the reactions in our model satisfied mass and charge balance using the checkMassCharge balance program from the COBRA Toolbox and balanced the unbalanced reactions, mostly by adding H_2_O or H^+^ as a reactant or product.

## Results and discussion

### Model statistics

We use two variants of our model: one for the minimal AM3 medium, and the other for the rich medium A. The first variant of our model corresponding to the minimal medium AM3 [3] includes finite uptake flux boundaries for only three amino acids (L-glutamate, L-cysteine and L-methionine) for which *A*. *succinogenes* is auxotrophic as determined experimentally by McKinlay et al. [6]. The other amino acid flux values are reduced to zero by accordingly adjusting the flux boundaries. The second variant of our model, corresponding to a rich medium (Medium A) containing yeast extract [3], includes finite uptake fluxes for all the amino acids present in the yeast extract. SBML versions of the models presented in this paper are included in the Supplementary Information, as described in Table A in S1 File.

**Fig 1:**
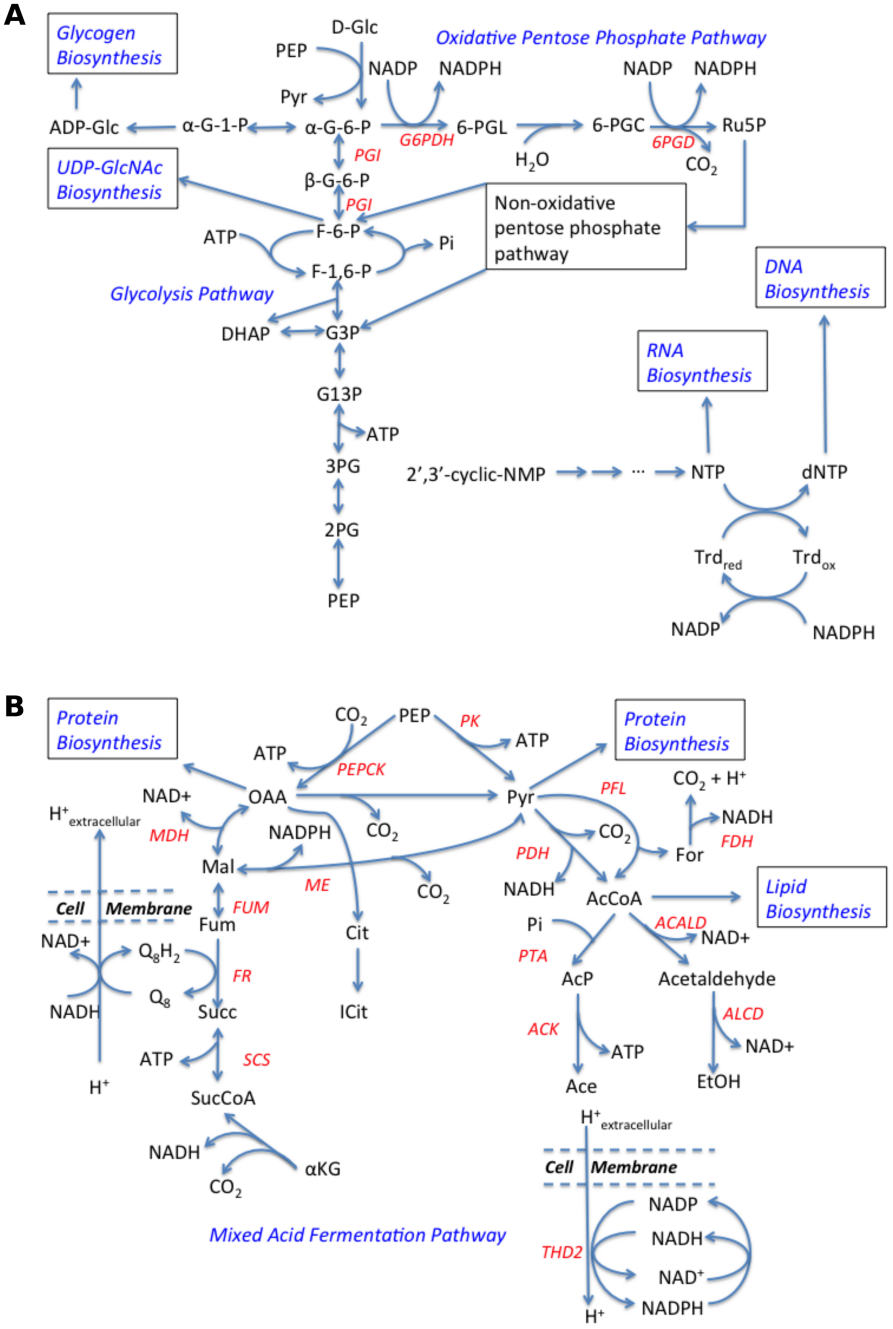
Schematic representation of the important pathways in model *A*. *succinogenes* metabolic network. NTP represents nucleotide triphosphate, where N = A, U, G, C. Part (a) shows the Glycolysis and the Oxidative Pentose Phosphate pathways, whereas part (b) shows the mixed acid fermentation pathway. The enzyme names are represented in red, whereas the pathway names are represented in blue.

The details of our model variants have been tabulated in Table B in S1 File. However, the most important features of our model can be summarized in the following schematic diagram (Fig 1).

#### Determination of stoichiometry in the biomass objective functio*n*

The objective function for our flux balance analysis is the flux through the pseudo-reaction for biomass synthesis (growth). The stoichiometry of the reactants and products of this pseudo reaction were estimated based mostly on experimentally determined values for *A*. *succinogenes* metabolic intermediate and cofactor requirements for biomass production (growth), as described below:

Amino Acids: The stoichiometries of the amino acids are estimated using the information on *A*. *succinogenes* metabolic intermediate and cofactor requirements for biosynthesis in Table 1 of McKinlay et al. [8].
*RNA and DNA*: We estimated the stoichiometric coefficients for RNA and DNA from Table 1 of McKinlay et al. [8]. However, the pseudo-reactions for the formation of RNA and DNA were taken from Table 1 of Knoop et al. [29] on the biomass objective function of *Synechocystis sp*. PCC 6803.
*Glycogen*: The glycogen metabolite in our model is actually the repeating monomeric unit (C_6_H_10_O_5_) in the linear glycogen polymer and has a molecular weight of 162.1406. This assumption, along with the percentage of dry cell weight of glycogen in Table 1 of McKinlay et al. [8] enabled us to calculate the stoichiometry of glycogen in the biomass formation reaction.
*Lipid*: In accordance with McKinlay et al. [8], the lipid composition is assumed to be 25% phosphatidylglycerol and 75% phosphatidylethanolamine. The *A*. *succinogenes* fatty acid composition (in percent of total lipid mass) has been measured by McKinlay et al. [8] as 14:0, 11%; 3-OH-14:0, 3%; 16:0, 35%; 16:1, 37%; C18:0,1%; C18:1, 3% and C18:2,10%. However, for the sake of simplicity, we included the biosynthesis of only palmitic acid (C16:0) in our model so that the only phosphatidylglycerol and phosphatidylethanolamine species in the model system are dipalmitoyl phosphatidylglycerol and dipalmitoyl-L-1-phosphatidylethanolamine respectively.
UDP-GlcNAc: UDP-N-Acetyl-α-D-glucosamine (UDP-GlcNAc) is an essential precursor of peptidoglycan (PG) and lipopolysaccharide (LPS). A close observation of Table 1 in [8] indicates that PG makes up 3.5% and LPS 4.7% of the dry cell weight respectively, so that PG and LPS collectively constitute 8.2% of the dry cell weight. We did not include the formation of polyamines in our central carbon metabolism model. Thus, if we add the contributions of all the biomass components, up to LPS, in the % of dry cell weight column of Table 1 in [8], we get 99.1% of the dry cell weight. To account for the remaining 0.9%, we attribute it to UDP-GlcNAc. Thus, attributing the 0.9% of the dry cell weight is a book keeping assumption that enables us to account for 100% of the dry cell weight. This assumption formed the basis for the estimation of the stoichiometry of UDP-GlcNAc in the biomass formation reaction.

#### Biomass objective function

The formation of biomass from these above-mentioned components was modeled as the following pseudo-reaction:

1.01663 Gly + 0.0857984 Tyr + 0.243293 Asp + 0.304278 Lys + 0.286834 Thr + 0.273954 Ile + 0.433024 Leu + 0.433769 Val + 0.160066 Arg + 0.245393 Asn + 0.232353 Glu + 0.234139 Gln + 0.236832 Pro + 0.0802104 His + 0.844144 Ala + 0.252649 Ser + 0.0872576 Cys + 0.083844 Met + 0.156278 Phe + 0.0322217 Trp + 0.438282 RNA + 0.142561 DNA + 0.407054 Glycogen + 0.0953814 dipalmitoyl-L-1-phosphatidylethanolamine + 0.030475 dipalmitoyl phosphatidylglycerol + 0.150329 UDP-GlcNAc + 46.93 ATP + 46.92 H_2_O-> 46. 93 ADP + 46. 93 Pi + 46.93 H^+^ + 1g biomass [gDCW^-1^],

where the stoichiometries of all the reactant/product species except biomass are in mmol. The flux through this pseudo-reaction constitutes the biomass objective function that is optimized for most of the constraint-based analyses discussed in the Results and Discussion section.

### Effect of varying bicarbonate uptake flux on *A*. *succinogenes* mixed acid fermentation

McKinlay et al. have undertaken ^13^C labeling experiments to investigate the fermentative metabolism in *A*. *succinogenes* grown in a chemically defined minimal medium AM3 with the medium NaHCO_3_ concentration varying from 5 to 150 mM. However, the fluxes for the cellular uptake of NaHCO_3_ and L-glutamate and the non-growth associated ATP requirement (NGAM) for A. succinogenes have not been estimated as part of the above-mentioned studies. We assumed a Michaelis-Menten relation between the medium bicarbonate concentration and the bicarbonate uptake flux into the cell. We thereby estimated the values of the maximal rate (V_max_) and the Michaelis-Menten constant (K_M_) for bicarbonate uptake, and the L-glutamate and non-growth associated ATP requirement for *A*. *succinogenes* in the AM3 medium by fitting model predictions to experimentally determined ratios of fermentation product secretion to glucose uptake fluxes. More precisely, this fitting is achieved by solving the following optimization problem: minimizing the objective function *F*, given by

$$F=\sum_{i}{\left[\frac{\left(\frac{x_{i}^{f,max}+x_{i}^{f,min}}{x_{glc}^{f,max}+x_{glc}^{f,min}})-(\frac{e_{i}}{e_{glc}}\right)}{\left(\frac{x_{i}^{f,max}}{x_{glc}^{f,min}}-\frac{x_{i}^{f,min}}{x_{glc}^{f,max}}\right)}\right]}^{2},$$

where *i* ∈ [succinate, formate, acetate, ethanol, growth]. In the above expression, $x_{i}^{f,min}$ and $x_{i}^{f,max}$ respectively denote the upper and lower bounds of the secretion flux of fermentation product *i* consistent with a fraction *f* of optimal growth, $x_{glc}^{f,min}$ and $x_{glc}^{f,max}$ represent the upper and lower bounds of the glucose uptake flux, and *e*_*i*_ and *e*_*glc*_ represent the experimentally estimated secretion flux of *i* and glucose uptake respectively. The flux values $x_{i}^{f,min}$, $x_{i}^{f,max}$, $x_{glc}^{f,min}$ and $x_{glc}^{f,max}$, which are determined using the flux variability analysis (FVA) functionality of COBRApy, are functions of the Michaelis-Menten parameters for bicarbonate uptake, the L-glutamate uptake flux and the NGAM for *A*. *succinogenes* and the objective function F is minimized with respect to these model parameters. For the fitting the glucose uptake into the cell was restricted between 5.9 and 6.2 mmol gDCW^-1^ hr^-1^, where the limits are obtained by averaging the lower and upper bounds of the experimentally estimated values of the glucose uptake flux. The value of the fraction *f* of the optimal growth that we have used to determine the flux ranges for the fitting is 96.5% since the flux ranges for fermentation product secretion corresponding to 96.5% of the maximum growth are the narrowest ones that completely contain the experimentally determined values of the fermentation product secretion fluxes. The values of the model parameters for *A*. *succinogenes* fermentative metabolism in AM3 medium are tabulated in Table C in S1 File.

**Fig 2:**
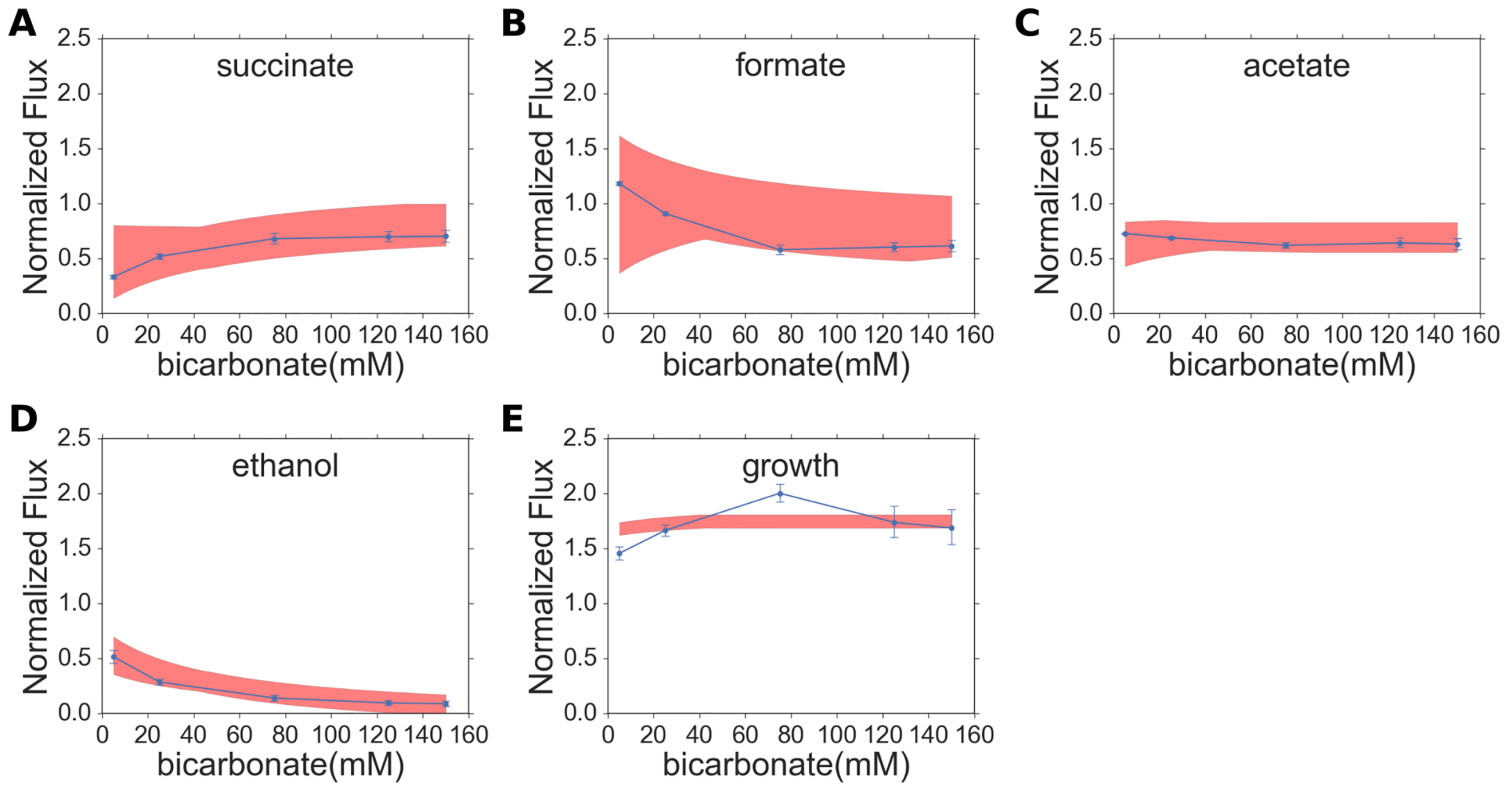
Comparison of experimentally derived fermentation product fluxes, normalized by glucose uptake with model predicted normalized fluxes for best-fit model parameters. The blue filled circles with error bars represent the experimental data points and the solid red line represents the midpoint of the FVA range, whereas the shaded regions represent the predicted FVA ranges corresponding to 96.5% of optimal growth.

The comparison of the experimental results and the model predictions corresponding to the best fit of the model parameters are presented in Fig 2. It is evident from Fig 2 that the succinate secretion flux increases with the bicarbonate uptake flux, whereas the formate and ethanol excretion fluxes reduce with increasing bicarbonate uptake flux, consistent with what has been observed in experimental studies on *A*. *succinogenes*.

Detailed experimental investigation by McKinlay et al. [4] indicate that the PEPCK flux was not affected by the NaHCO_3_ concentrations in the medium. Increasing the NaHCO_3_ concentration in the medium, and hence the CO_2_ uptake rate, causes the reverse flux through the reaction catalyzed by malic enzyme to preserve mass balance. As a result, the net flux through the malic enzyme reaction decreases. On the basis of their experimental results, McKinlay et al. postulated that decreasing malate decarboxylating flux and constant flux through the PEPCK reaction with increasing medium bicarbonate concentration resulted in increased flux through the C4 pathway and hence increased succinate production [4].

**Fig 3:**
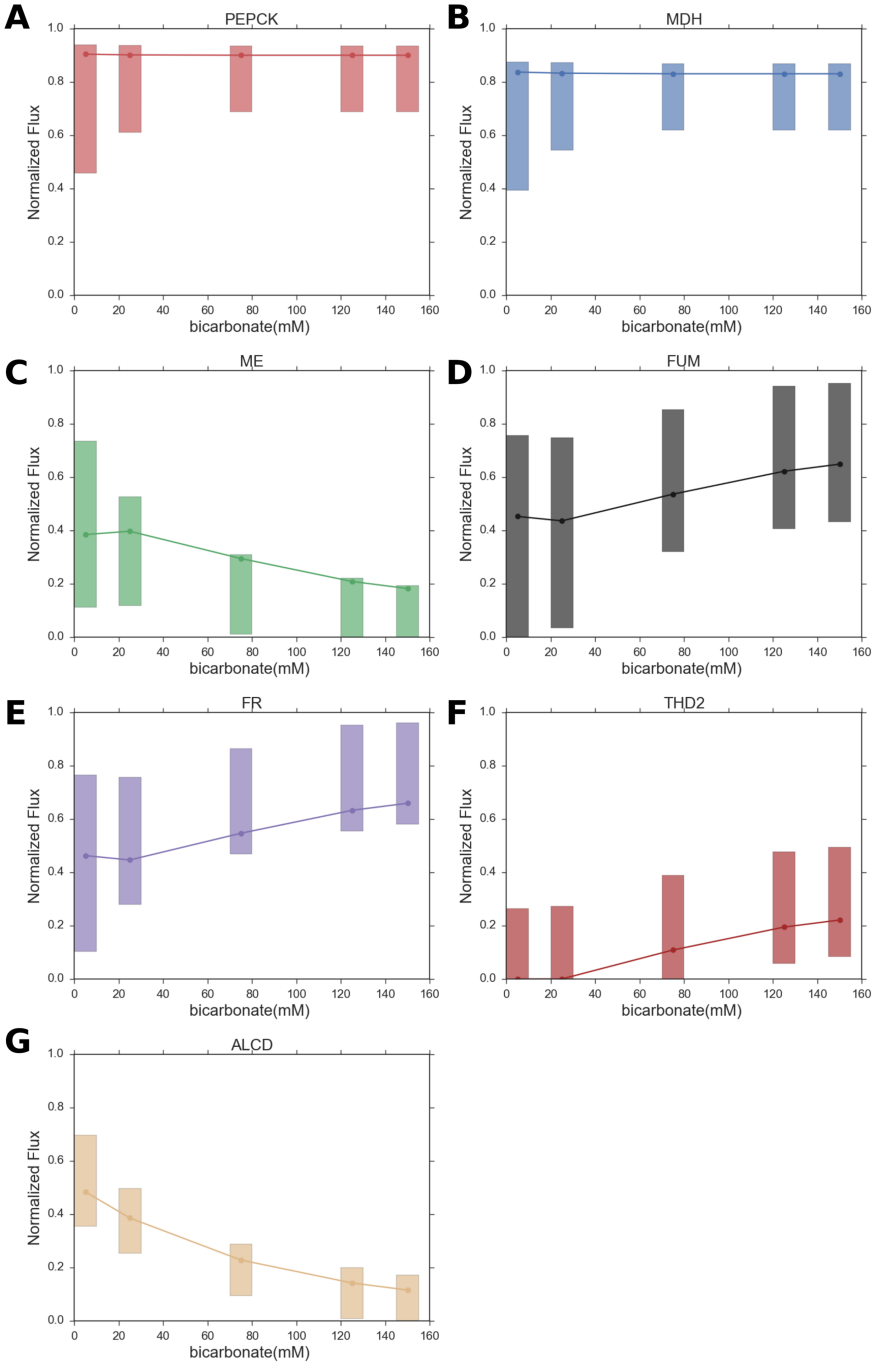
Model predicted optimized fluxes (from pFBA) and flux ranges (from FVA) for relevant fluxes in the *A*. *succinogenes* mixed acid fermentation pathway. (a) PEPCK (phosphoenolpyruvate carboxykinase), (b) MDH (malate dehydrogenase), (c) ME (malic enzyme), (d) FUM (fumarase), (e) FR (fumarate reductase), (f) THD2 (transhydrogenase), and (g) ALCD (alcohol dehydrogenase).

We have used parsimonious flux balance analysis (pFBA) [30] and flux variability analysis (FVA) [31] to predict parsimonious solutions and flux ranges respectively for several reactions in the *A*. *succinogenes* fermentation pathway with increasing medium bicarbonate concentration. Since bicarbonate uptake follows Michaelis-Menten kinetics, increasing medium bicarbonate concentration translates to increasing bicarbonate uptake flux. The model predictions for the reactions PEPCK (phosphoenolpyruvate carboxykinase), MDH (Malate dehydrogenase), ME (Malic Enzyme), FUM (Fumarase), FR (fumarate reductase), THD2 (transhydrogenase) and ALCD (alcohol dehydrogenase), shown schematically in Fig 1, are presented in Fig 2. It is evident from Fig 3, that according to our model, the carbon flux in the forward direction of ME decreases with increasing medium bicarbonate concentration. On the other hand the carbon flux from phosphoenolpyruvate to L-malate via the PEPCK and MDH reactions, shown in Fig 1b, remains unchanged as predicted by McKinlay et al. [4]. Hence, as evident from the schematic in Fig 1b, in order to maintain mass balance, the carbon flux from malate to succinate, via fumarate, has to increase.

The gradual decrease of the reaction flux in the forward direction of ME with increasing bicarbonate uptake can be interpreted as increasing flux in the reverse direction of the ME (see Fig 1b). This change leads to increased demand for NADPH, which is achieved by increasing the flux through the THD2 reaction (Fig 1b), which in turn requires NADH. The increasing demand for NADH is satisfied by gradual reduction of flux through the ALCD reaction, which utilizes NADH (Fig 1b). A careful observation of Fig 3 indicates that the increases in flux between successive medium bicarbonate concentrations for FUM, FR and THD2 roughly equal the decreases in flux between successive medium bicarbonate concentrations for ME and ALCD. Thus, our model provides an explanation, at least on a qualitative level, about the variation trends of several vital fermentation pathway fluxes with increasing bicarbonate uptake as postulated on the basis on experimental results.

### Comparison of *A*. *succinogenes* growth and mixed acid fermentation in minimal and rich growth media

In addition to experiments in the AM3 medium, McKinlay also undertook an experimental investigation to compare the growth trends and fermentation balances of *A*. *succinogenes* in minimal medium AM3 and a rich medium known as Medium A [3]. For this study the NaHCO_3_ concentration in both AM3 and Medium A was taken to be 150 mM. In medium A, the vitamins, minerals, amino acids, NaCl, and NH_4_Cl in AM3 are substituted by 5g/liter of yeast extract. We found a detailed account of the molecular composition of yeast extract from Smith et al. [32].

Assuming that the parameters in Table C in S1 File will not vary between the minimal medium AM3 and the rich medium Medium A, we have predicted the ranges of fermentation product excretion fluxes corresponding to 96.5% of the optimal growth for *A*. *succinogenes* grown in the rich Medium A. The predictions are made for a glucose uptake rate of 8.65 mmol gDCW^-1^ hr^-1^ and a medium carbonate concentration of 150 mM in agreement with the experimentally derived fluxes for the rich medium A.

**Fig 4:**
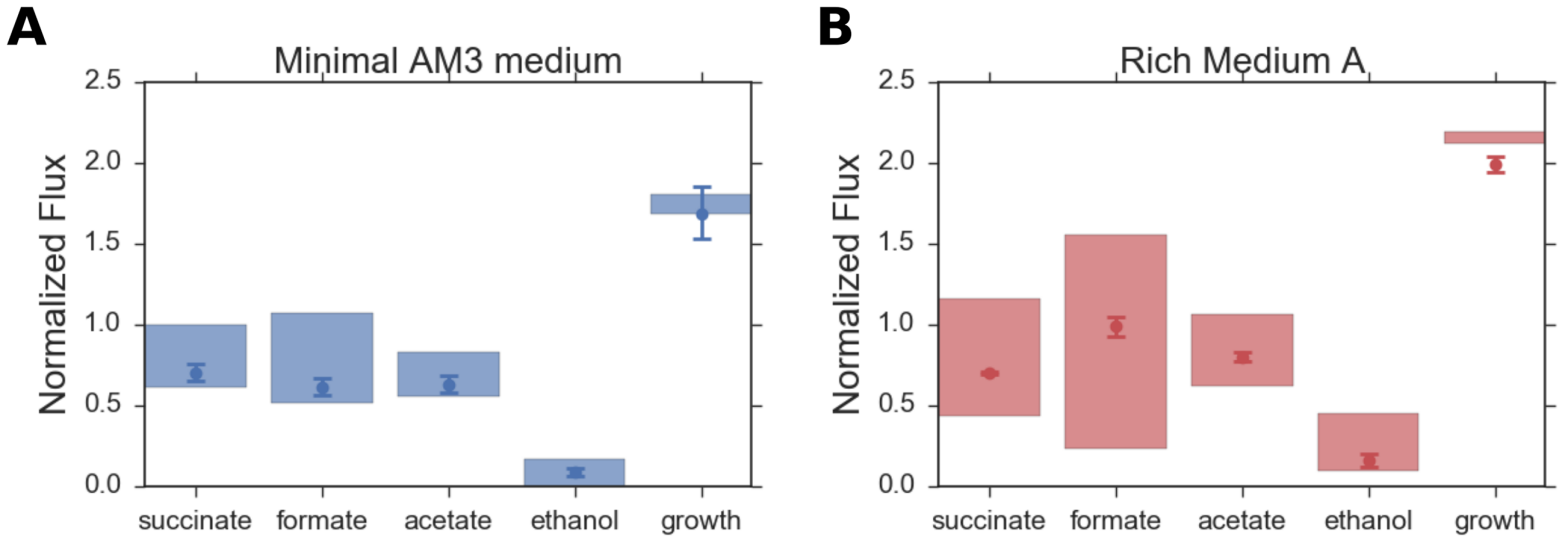
Comparison of model-based predictions of growth and flux ranges to experimental results for *A*. *succinogenes* grown in (a) rich medium A and (b) minimal AM3. Shaded regions correspond to flux ranges predicted for 96.5% of optimal growth. Circles and corresponding error bars represent experimentally derived fermentation product fluxes. Both predicted ranges and experimental fluxes are normalized by glucose uptake, with a NaHCO3 concentration 150 mM in both media.

In Fig 4, we have compared the predictions of the model for the minimal AM3 medium as well as for the rich medium A, with the corresponding experimental results. It is clearly evident from Fig 4a and 4b that the model-predicted flux ranges for the fermentation products contain the experimentally estimated values and the predicted growth shows qualitative agreement with the experimentally derived growth as well. A comparison of the two parts of Fig 4 indicates that our model correctly predicts that the succinate production flux is similar in the two media, whereas the formate, acetate and ethanol production and the growth fluxes are higher in the rich medium A compared to the minimal medium AM3.

We also assessed the effect of deletion of each gene and each reaction in the models for both the minimal (AM3) and rich (medium A) media, for a medium bicarbonate concentration of 150 mM. Both FBA and minimization of metabolic adjustment (MOMA) methods were used for these simulations but the results were identical for both the methods as evident from the Table 1.

**Table 1:** Lethality of single gene and reaction deletions.

	Medium A (Rich)		AM3 (Minimal)	
	FBA	MOMA	FBA	MOMA
**Number of lethal reactions**	48	48	118	118
**Number of lethal genes**	40	40	97	97

Both the numbers of lethal gene and reaction deletions were lower in the rich medium compared to the minimal media. The rich medium (Medium A) contains yeast extract, so it already contains most of the amino acids [32]. Hence, several reactions in the amino acid biosynthetic metabolism and related genes that are essential for AM3 are no loner essential for Medium A. This causes lower number of lethal reactions and genes for Medium A compared to AM3. The lists of the lethal reactions for both the minimal and the rich media are provided in MS Excel files referred to in Table D in S1 File.

### Growth of *A*. *succinogenes* on L-glutamate and various L-glutamate precursors

McKinlay et al. have experimentally examined whether glutamate precursors support growth of *A*. *succinogenes* in the AM3 medium [3]. Using the parameters in Table C in S1 File, we determined if our model could be used to make similar predictions about growth on these different glutamate precursors. The uptake flux of L-glutamate or its precursor (L-glutamine, α-ketoglutarate, aspartate) was set to the fitted value for L-glutamate uptake flux in Table C in S1 File. Table 2 shows that predictions from our model agree with experimental predictions on the growth capability of *A*. *succinogenes* on various precursors of L-glutamate.

**Table 2:** Metabolic model based prediction of L-glutamate precursors to support growth of *A*. *succinogenes* in AM3 medium.

Glutamate precursor	Observed Growth^a^	Predicted Growth^b^
**NH_4_^+^**	-	0
**NH_4_^+^ + Glu**	+	0.268
**NH_4_^+^ + α-ketoglutarate**	+	0.27
**Gln**	+	0.215
**Asp**	-	0
**Asp + α-ketoglutarate**	+	0.195

^a^ Experimentally observed growth from McKinlay et al. [3];

^b^ Model-predicted growth flux (gDCW hr^-1^) from FVA

### Effect of varying both bicarbonate and hydrogen uptake fluxes on fermentative metabolism

McKinlay et al. [4] also explored the fermentative metabolism of *A*. *succinogenes* at different NaHCO_3_ and H_2_ concentrations using ^13^C metabolic flux analysis. For this investigation, they used a variant of the AM3 medium in which the Cys–HCl, Met, and monosodium glutamate concentrations were changed relative to the minimal AM3 medium [4]. McKinlay and co-workers used two different concentrations of NaHCO_3_ in this growth medium, 25 mM and 100 mM, both in the presence and absence of H_2_. For medium NaHCO3 concentrations of 25 and 100 mM, the H_2_ oxidation rate were measured as 2.8 mmol gDCW^-1^ hr^-1^ and 4.7mmol gDCW^-1^ hr^-1^ respectively. The experimentally estimated glucose uptake rate varied between 9 and 10 mmol gDCW^-1^ hr^-1^ for the above-mentioned medium. In our model, we have assumed that the H_2_ input fluxes equal the H_2_ oxidation rates in McKinlay et al. [4]. Using the parameters in Table C in S1 File, we predicted the growth and fermentation product fluxes for 4 different cases: low and high bicarbonate concentration, and low and high H_2_ concentration. Since the energy and redox cofactor yield of different fermentation product pathways are similar, the ranges of predicted product fluxes are large in comparison with the experimental error. These ranges therefore represent all stoichiometrically feasible growth modes capable of yielding a similar biomass growth rate.

**Fig 5:**
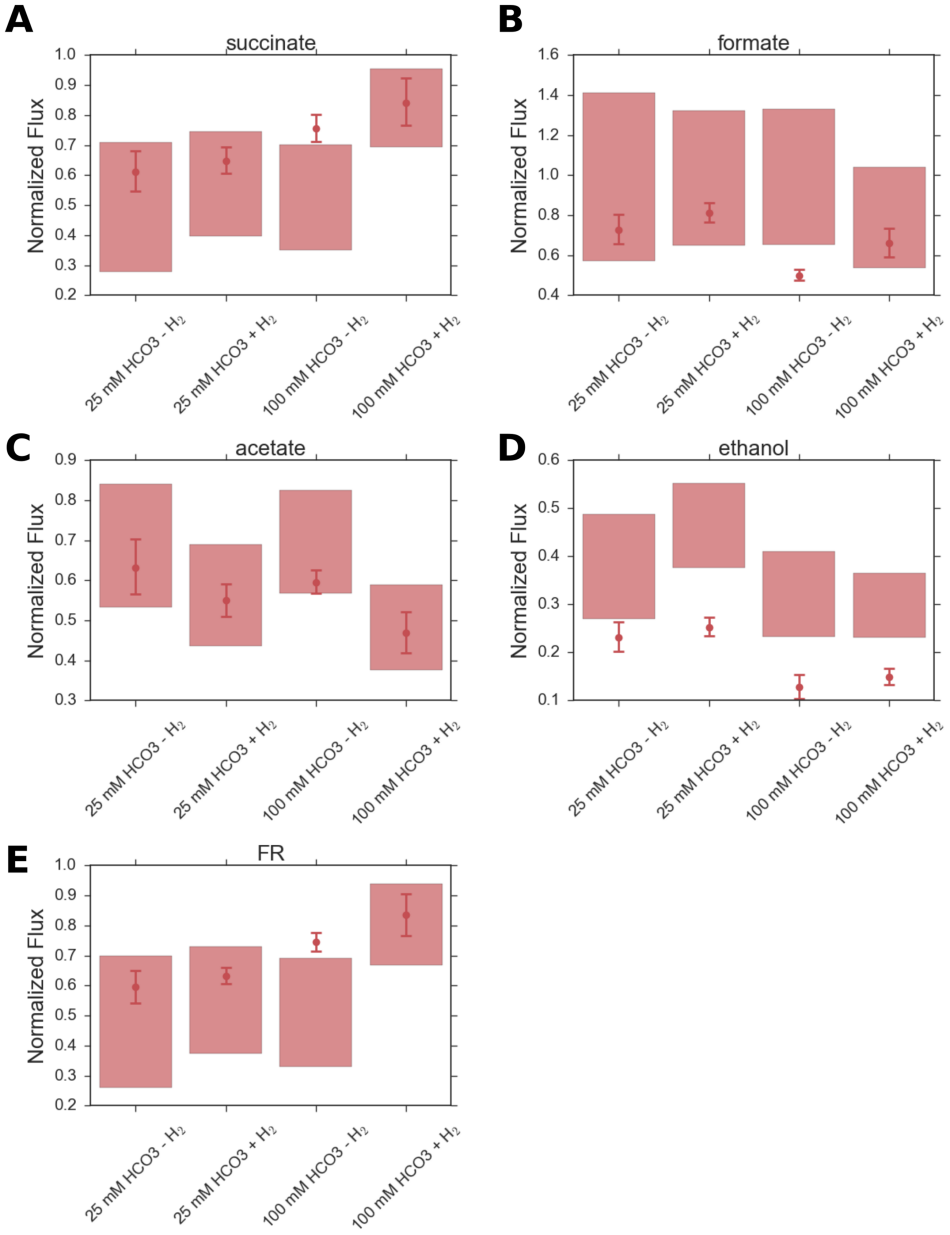
Flux predictions for different hydrogen and bicarbonate media conditions. Prediction of fermentation end product secretion, growth and fumarate reductase (FR) fluxes for two different medium bicarbonate concentrations (25 mM and 100 mM) and with/without H_2_. Shaded regions correspond to flux ranges predicted for 96.5% of optimal growth by FVA (flux variability analysis). Circles and corresponding error bars represent experimentally derived fermentation product fluxes.

As shown in Fig 5, the model predicts that growing *A*. *succinogenes* in the presence of H_2_ results in higher succinate excretion and this effect is accentuated at higher bicarbonate uptake fluxes, which agrees qualitatively with the experimentally derived results [4]. McKinlay et al. [4] have suggested that the flux through the FR reaction (Fig 1b) is limited by the availability of reductants in the absence of additional electron sources. When H_2_ is available as an electron source, additional reducing equivalents are available so that the flux through the FR reaction increases, resulting in higher succinate formation. It is evident from Fig 5e that our model correctly predicts the increase in the FR flux in the presence of H_2_. Not only that, in accordance with the experimental results [4], our model also predicts that the increase in FR flux in the presence of H_2_ is more pronounced at the higher bicarbonate concentration than that at the lower bicarbonate concentration. It should be noted that though our model consistently overestimates the ethanol formation fluxes at various medium bicarbonate concentrations and with/without hydrogen uptake, it qualitatively reproduces the trend for ethanol formation as a function of these media conditions. Experimental papers on *A*. *succinogenes* metabolism [3, 4, 6, 8] seem to indicate that the overall formation of ethanol from acetyl-CoA is irreversible and this is what we have assumed in our model as well. However, the pathway-genome databases for *A*. *succinogenes* in KEGG and MetaCyc seem to indicate a degree of reversibility of the reaction from acetaldehyde to ethanol in Fig 1. According to the pathway-genome database for *A*. *succinogenes* in MetaCyc, ethanol can be degraded to acetate via acetaldehyde. The presence of such a degradation pathway *in vivo*, which we do not consider *in silico*, might be the cause behind the overestimation of the ethanol production flux by our model.

### Prediction of reaction knockouts to maximize succinate production

We used the OptKnock algorithm to predict both single and double reaction knockouts for maximizing succinate production in the minimal AM3 medium while restraining the growth to be at least 0.1 gDCW hr^-1^ and the non-growth associated ATP maintenance flux to be at 1.082 mmol gDCW^-1^ hr^-1^. A subset of model reactions was selected for knocking out that excluded the ATP synthase, NGAM, and the uptake and excretion reactions.

Depending on whether we predicted single or double reaction knockouts, we compared the OptKnock results with results from flux variability analysis, after knocking out one or a pair of reactions from the above-mentioned subset. However, in the case of *in silico* double mutants, instead of running the FVA for all possible pairs of reactions from the set selected for knockouts, we created a set of reactions corresponding to the top FVA predictions for single reaction deletions. We then chose all possible reactions pairs from this latter set and ran the FVA after *in silico* deletion of each possible reaction pair.

For the minimal AM3 medium, our estimate for the L-glutamate flux was about 0.439 mmol gDCW^-1^ hr-^1^. However, the L-glutamate flux for knockout strains might vary over a range and we chose the range of L-glutamate fluxes from the robustness analysis of *A*. *succinogenes* growth with respect to L-glutamate uptake flux. Hence, the extended range of L-glutamate uptake fluxes that we considered based on robustness analyses was 0–0.5 mmol gDCW^-1^ hr-^1^. Similarly, the range of bicarbonate fluxes chosen for the reaction knockout study was chosen as 0–5.0 mmol gDCW^-1^ hr-^1^. The single knockouts that are most effective in maximizing succinate production involve knocking out the reactions catalyzed by the PTA (Phosphate acetyltransferase) and ACK (Acetyl kinase) enzymes. The double knockouts that are most effective in increasing succinate production are the (PEPCK, PTA) and (PEPCK, ACK) knockouts.

Knocking out the reaction catalyzed by PTA or ACK reduces the carbon flux from pyruvate to acetyl-CoA since the conversion of acetyl-CoA to acetate is favored over the conversion of acetyl-CoA to ethanol, as the latter route requires more reducing equivalents (Fig 1b). For the PTA or ACK knockout strain, it is predicted by our model that the lower flux from pyruvate to acetyl-CoA is compensated for by a net flux in the reverse direction of the malic enzyme catalyzed reaction, resulting in higher succinate excretion via higher FUM and FR fluxes (Fig 1b). However, it should be noted that additional reducing equivalents (NADPH) are required for the reverse direction of the malic enzyme reaction. Our model predicts that for the wild type strain there is practically no flux through the oxidative branch of the pentose-phosphate pathway and that carbon flow from glucose -6-phosphate (G6P) occurs almost exclusively to fructose-6-phosphate (F6P) via the PGI (glucose-6-phosphate isomerase) reaction (Fig 1a). On the contrary, for the PTA or ACK knockout strain, the reaction flux from glucose-6-phosphate through the oxidative pentose phosphate pathway roughly equals the reaction flux from G6P to F6P. The carbon flux through the oxidative pentose phosphate pathway yields additional NADPH via the G6PDH (glucose-6-phosphate dehydrogenase) and 6PGD (6-phosphogluconate dehydrogenase) reactions, as shown in Fig 1a. This additional NADPH enables a net positive reaction flux in the reverse direction of the malic enzyme reaction from pyruvate to malate.

For the predicted double mutants (PEPCK, PTA) and (PEPCK, ACK), the reaction flux in the PEPCK reaction from phosphoenolpyruvate to oxaloacetate in the wild type and single knockout mutants is diverted to the PK (pyruvate kinase) reaction (Fig 1a). There is no flux from pyruvate to acetyl-CoA via the PFL (pyruvate formate lyase) and PDH (pyruvate dehydrogenase) reactions. Instead, there is a net flux from pyruvate to malate via the reverse direction of the ME (Fig 1b). This flux is larger than the corresponding flux in the *in silico* single reaction knockouts. Additional NADPH required for the increased flux in the reverse direction of the malic enzyme reaction is made available by splitting the flux from β-glucose-6-phosphate to both the oxidative branch of the pentose phosphate pathway and to β-glucose-6-phosphate via the PGI reaction (Fig 1b), with a greater fraction of the flux being directed towards the oxidative pentose phosphate pathway. Also, there is no net flux in the forward direction of the MDH reaction, which utilizes NADH (Fig 1b). Instead there a small flux from malate to oxaloacetate via the reverse direction of the MDH reaction, which is required to generate the essential amino acid L-aspartate from oxaloacetate. The reverse direction of the MDH reaction, instead of consuming NADH, yields NADH. This additional NADH is converted to NADPH via the THD2 reaction.

It should be noted that both the single and double reaction knockouts cause increase in succinate flux without stalling the biomass production or growth flux. Thus, for the single ACK or PTA knockouts, the model predicted growth flux is reduced to about 84.5% of the predicted growth flux for the *in silico* WT strain, whereas for the double knockouts (ACK, PEPCK/PTA, PEPCK) the predicted growth flux is 74.9% of the predicted growth flux for the *in silico* WT strain. This observation is indicative of the robustness of our model system.

## Conclusions

We have developed a detailed metabolic model for the gram-negative succinogenic bacterium, *A*. *succinogenes*. This model contains most of the pathways relevant to the biosynthesis of the main components of biomass, namely glycogen, proteins, lipids, DNA and RNA and UDP-GlcNAc. We have validated this model by comparing the model predictions with results and postulations from published reports on experimental investigation of the mixed acid fermentation in *A*. *succinogenes*. We then use variants of this model corresponding to different growth media to make predictions about single and double reaction knockouts to optimize succinate production.

On the modeling and computational front, future work using this model would involve extending the model to the genome scale and examine if the model extension causes model predictions to vary. In a different direction, it would be worthwhile to work towards experimentally executing the knockouts predicted by the model. In an ideal situation, these two directions would go hand in hand and form different steps of a combined and iterative computational and experimental workflow where the model helps to predict knockouts and the experimental validation or invalidation of the predicted knockouts helps to refine or extend the model.

## Supporting information

S1 File Tables A-E. (DOCX)

S1 Model Model file for minimal AM3 and modified AM3 media. (XML)

S2 Model Model file for medium A (rich medium). (XML)

S1 Data Set Detailed list of essential reactions for minimal AM3 medium. (XLSX)

S2 Data Set Detailed list of essential reactions for medium A (rich medium). (XLSX)
