# Supplementary material for: Prediction of reaction knockouts to maximize succinate production by Actinobacillus succinogenes
Source: PLoS One. 2018 Jan 30;13(1):e0189144. doi: 10.1371/journal.pone.0189144 (PMC5790215; doi:10.1371/journal.pone.0189144)
Supplement: S1 File — (DOCX) [file pone.0189144.s001.docx]

**Supplementary Information**

Table A: Model files for different media

| **Medium** | **Model file** |
| --- | --- |
| Minimal AM3 | S1 Model.xml |
| Modified AM3 | S1 Model.xml |
| Medium A (rich medium) | S2 Model.xml |

Table B: Model statistics

| Model File | S1 Model.xml | S2 Model.xml |
| --- | --- | --- |
| Number of Metabolites | 263 | 263 |
| Number of Reactions | 360 | 375 |
| Number of Boundary Fluxes | 47 | 62 |
| Number of Genes | 272 | 272 |

Table C: Fitted model parameters for *A. succinogenes* metabolic model

| **Parameter** | **Fitted Value** | **Unit** |
| --- | --- | --- |
| Bicarbonate uptake *V_max_* | 4.806 | mmol gDCW^-1^hr^-1^ |
| Bicarbonate uptake *K_M_* | 45.773 | mM |
| L-glutamate uptake | 0.439 | mmol gDCW^-1^hr^-1^ |
| NGAM | 1.082 | mmol gDCW^-1^hr^-1^ |

Table D: Detailed list of essential reactions for various media

| **Medium** | **File** |
| --- | --- |
| Minimal AM3 | S1 Data Set.xlsx |
| Medium A (rich medium) | S2 Data Set.xlsx |

Table E: Mapping between reaction IDs in the model files and enzyme names and abbreviations used in the manuscript.

| **Model Reaction** | **Description** | **Abbreviation** |
| --- | --- | --- |
| MNXR2456_i | Glucose-6-phosphate dehydrogenase | G6PDH |
| MNXR2793_i | 6-Phosphogluconate dehydrogenase | 6PGD |
| MNXR35757_i | Phosphoglucoisomerase | PGI |
| MNXR6501_i | Phosphoglucoisomerase | PGI |
| MNXR4383_i | Phosphoenolpyruvate carboxykinase | PEPCK |
| MNXR4545_i | Pyruvate kinase | PK |
| MNXR4175_i | Pyruvate formate lyase | PFL |
| MNXR4132_i | Pyruvate dehydrogenase | PDH |
| MNXR2200_i | Formate dehydrogenase | FDH |
| MNXR3464_i | Malate dehydrogenase | MDH |
| MNXR3471_i | Malic Enzyme | ME |
| MNXR2338_i | Fumarase | FUM |
| MNXR55430a_i | Fumarate reductase | FR |
| MNXR4958_i | Succinyl-CoA synthetase | SCS |
| MNXR4502_i | Phosphate acetyltransferase | PTA |
| MNXR390_i | Acetyl kinase | ACK |
| MNXR353_i | Acetaldehyde dehyrogenase | ACALD |
| MNXR648_i | Alcohol dehydrogenase | ALCD |
| MNXR3771a_i | NAD(P) transhydrogenase | THD2 |
